# Role of Antibiotic Prophylaxis in the Management of Antenatal Hydronephrosis, Vesicoureteral Reflux, and Ureterocele in Infants

**DOI:** 10.7759/cureus.9064

**Published:** 2020-07-08

**Authors:** Sadaf Faiz, Mitul P Zaveri, Jamal C Perry, Tayná M Schuetz, Ivan Cancarevic

**Affiliations:** 1 Medicine, California Institute of Behavioral Neurosciences & Psychology, Fairfield, USA; 2 Internal Medicine, California Institute of Behavioral Neurosciences & Psychology, Fairfield, USA; 3 Research, California Institute of Behavioral Neurosciences & Psychology, Fairfield, USA

**Keywords:** antibiotic prophylaxis, antenatal hydronephrosis, vesicoureteral reflux, ureterocele, urinary tract infection

## Abstract

Widespread prenatal screening has resulted in increased detection of anomalies of the kidneys and urinary tract. Antenatal hydronephrosis (AHN) and vesicoureteral reflux (VUR) are among the most common congenital anomalies diagnosed in utero or after birth. Pediatric urologists frequently rely on continuous antibiotic prophylaxis (CAP) for managing AHN, VUR, and ureterocele, unless definitive treatment is performed. The main aim of antibiotic prophylaxis (ABP) is to prevent urinary tract infection and long-term complications. Nevertheless, the efficacy of ABP has been a source of considerable debate, and pediatricians have varied opinions on who would benefit from ABP. In this review article, we searched the currently available literature, for evidence of the role of ABP in the setting of AHN, VUR, and ureterocele. Most of our studies showed a limited benefit of ABP for HN and VUR. The data on the use of CAP in the management of ureterocele is scarce. However, due to the involvement of independent risk factors and other variables, a conclusion cannot be drawn from these studies alone. Pediatric urologists are urged to conduct randomized controlled trials to compare patients followed up with and without ABP. Given the lack of guidelines, an individualized approach should be used for the use of ABP, until precise guidelines and recommendations are developed.

## Introduction and background

A congenital anomaly is an inherited medical condition that occurs at or before birth. It can be a structural, functional, or metabolic anomaly that originates during intrauterine life and can interfere with the body functions. These anomalies are a significant cause of chronic illnesses, disability, and death in children [1]. The congenital anomalies of the kidney and urinary tract (CAKUT) form a group of heterogeneous disorders that affect the kidneys, ureters, and bladder. These anomalies include, among others, renal agenesis, renal hypoplasia, multicystic dysplastic kidney, hydronephrosis (HN), ureteropelvic junction obstruction, megaureter, ureter duplex, vesicoureteral reflux (VUR), ureterocele, and posterior urethral valves [2]. In young children, CAKUT are the main cause of the end-stage renal disease (ESRD), leading to the need for kidney transplantation or dialysis [3]. The survival rate of this group is 30 times lower than that of healthy children [3]. It may also be associated with kidney problems later in life, such as hypertension, proteinuria, and ESRD [4].

The widespread use of routine second-trimester ultrasounds has increased the detection of congenital anomalies. Antenatal hydronephrosis (AHN) is one of the most common anomalies detected on routine prenatal ultrasounds, affecting 1%-4.5% of all pregnancies [5]. It represents approximately 30% of all congenital defects detected on antenatal scans [6]. A variety of urological conditions can present with AHN, and differential diagnosis can usually be made only after thorough postnatal assessment [6]. The most common postnatal diagnoses include primary VUR in 10%-20% of cases, nonrefluxing HN (caused by ureteropelvic junction obstruction) in 10%-30% of cases, primary nonrefluxing megaureter in 5%-10% of cases, and ureterocele in 5% of cases [6]. HN is a condition characterized by an excessive amount of urine inside the pelvicalyceal system, leading to its dilation and distension [7]. The prevalence is higher in boys, and it is most commonly diagnosed in children younger than one year of age [8]. Due to impaired drainage or VUR, urinary stasis may develop in patients with hydronephrosis, leading to an increased risk of urinary tract infections (UTIs) [9]. Hydronephrosis is diagnosed by ultrasonography; its outcome and management largely depends on the underlying etiology and severity.

VUR is the retrograde flow of urine from the bladder to the upper urinary tract (i.e., into one or both ureters, and then to renal calyx or kidneys). It is an important risk factor for renal scarring in children who present with UTI [10]. There is an overall high prevalence of reflux in children younger than five years of age diagnosed with UTI [10]. The incidence is even higher in infants younger than one-year-old (i.e., as high as 70%) [10]. Developmentally, VUR can be classified into two types, namely, primary and secondary. Primary VUR is the more common type and occurs through a defective ureterovesical junction (UVJ) [11]. Causes of secondary VUR include functional (e.g., bladder or bowel dysfunction and neurogenic bladder) and anatomic outlet obstruction (e.g., posterior urethral valve) [10-11]. VUR is graded depending on the degree of dilatation and renal collecting system morphology into five grades, grade I being mildest (i.e., into a non-dilated ureter) and grade V being the most severe (i.e., the gross dilation of ureter, pelvis, and calyces with loss of papillary impressions and ureteral tortuosity) [12]. VUR is diagnosed by voiding cystourethrogram (VCUG), and the management depends on the grading and underlying cause [10].

A ureterocele is a rare form of CAKUT and is characterized by a cystic dilatation of the terminal ureter within bladder, urethra, or both [4]. The incidence shows a wide range from 1/500 to 1/12000 in the literature, and it is four to six times more common in females [13]. It is mostly found in children younger than two years of age [13]. They are classified as intravesical or ectopic based on their location [14]. The intravesical ureteroceles are located in the bladder trigone, and the ectopic forms are found in the bladder neck or posterior urethra [14]. Children with ureteroceles can present in different ways, from the asymptomatic patient with an antenatally detected ureterocele, to those with life-threatening urosepsis [15]. However, the most common presentation in young as well as older pediatric populations is a UTI [15]. Most cases of ureterocele are associated with hydroureteronephrosis, VUR, and complete ureteral and renal duplication (duplex kidney) [15].

The majority of pediatric urologists opt for continuous antibiotic prophylaxis (CAP) for managing AHN, VUR, and ureterocele, before the surgical intervention or until the resolution of symptoms. However, their role in the treatment remains controversial, and the studies are sparse in this setting. The prophylactic policies have turned out to be extremely variable for each condition, as the UTI rates widely vary between patients followed on and off antibiotics [6]. This comprehensive review highlights the role of antibiotic prophylaxis (ABP) in the management of AHN, VUR, and ureterocele in infants. We will discuss studies showing the benefit, or no benefit of CAP in each disease. We will also briefly review the risks of long-term use of prophylactic antibiotics.

## Review

Discussion

CAP has conventionally been offered to children with recurrent UTIs or those at risk, including children diagnosed with AHN, VUR, ureterocele, and other CAKUT. UTIs in the pediatric population are associated with long-term complications, including hypertension, renal insufficiency, renal scarring, and growth impairment [16]. Newborns with AHN have been shown to have a twelve-fold higher risk of hospitalization, predominately in the first year of life, and VUR accounts for approximately 10%-15% of children with prenatal HN [17]. Both hydroureteronephrosis and VUR are considered risk factors for febrile UTIs [14]. According to a study by Visuri et al., infants with AHN and grade IV-V VUR had the highest risk of UTI, and the UTIs tended to be more common in females than in males; however, males experienced UTI at a younger age than females [18]. It is believed that incomplete bladder emptying facilitates an environment for bacterial proliferation, and that the VUR allows the retrograde flow of infected urine into the upper urinary tracts leading to pyelonephritis and subsequent renal scarring [9]. The use of prophylactic antibiotics, gender, degree of kidney dilation, and circumcision status all have been reported to have some level of impact on UTIs [19]. Recurrent UTIs are observed in 30%-50% of children after the first UTI [20]. Of these, approximately 90% occurs within three months of the initial episode [20]. The bacteria most frequently responsible are Gram-negative organisms, with Escherichia coli accounting for 80% of urinary tract pathogens [20]. Because younger children and infants often have vague symptoms, the physician must have a high index of suspicion for UTI. The basic aim of ABP in children with urinary tract anomalies is the reduction of UTI frequency [20]. Many trials have been conducted for establishing the efficacy of CAP in the setting of HN, VUR, ureterocele, and other anomalies.

Hydronephrosis and Antibiotic Prophylaxis

ABP has been empirically recommended for newborns with ANH in an attempt to reduce the rate of UTIs in the first two years of life. Braga et al. did a meta-analysis including 3876 infants [21]. It was demonstrated that neonates with high-grade HN receiving antibiotic prophylaxis have a significantly lower rate of UTI when compared to untreated neonates (14.6% vs. 28.9%; p < 0.01) [21]. Rates of UTIs in patients with low-grade HN were similar, regardless of CAP status (i.e., 2.2% on prophylaxis vs. 2.8% not receiving prophylaxis) [21]. Herz et al. did a study in which they collected records of children referred to their institution for ANH from the year 2001 to 2011 [22]. Among the 405 children (236 girls and 169 boys; average age 2 days to 5.8 months) meeting the inclusion criteria, 278 (i.e., 68%) were maintained on CAP (YCAP group), and 127 (i.e., 31.4%) were not given CAP (NCAP group) [22]. The overall incidence of febrile UTI was 22.2% during the follow-up period, and was found to be significant between the YCAP and NCAP groups (i.e., YCAP = 7.9% and NCAP 18.7%, p = 0.021) [22]. It was concluded from the study that CAP reduces the risk of febrile UTI in children having independent risk factors (i.e., high-grade VUR, ureterovesical junction obstruction, and ureteral dilation) with ANH [22]. Varda et al. did a retrospective cohort study on 500 newborns with a history of antenatal urinary tract dilation (AUTD), in which six were excluded due to concomitant diseases [23]. Baseline patient (sex, race, insurance) and clinical characteristics were collected via retrospective chart review [23]. Among the 494 infants having AUTD, 157 (32%) received CAP [23]. There was no difference in CAP based on sex, insurance, or circumcision status among 260/365 males with known circumcision status [23]. Overall, seven infants developed UTI prior to imaging: six (1.8%) without CAP vs. one (0.64%) with CAP (p = 0.44) [23]. The authors concluded that the routine use of CAP in newborns with AUTD prior to initial imaging may be of minimal benefit in most patients [23]. In an analysis by Zareba et al., 376 infants (277 males and 99 females) with prenatal HN were identified in an institutional database [24]. HN was high-grade in 128 infants (34.0%) and VUR was present in 79 (21.0%) [24]. ABP was prescribed in 60.4% of patients, preferentially to females versus males (70.7% vs. 56.7%), those with high versus low-grade HN (70.3% vs. 55.2%) and those with versus without VUR (96.2% vs. 50.8%) [24]. On multivariate analysis, a link was found between high-grade HN and an increased risk of UTI; also, females and uncircumcised males were at higher risk than circumcised males [24]. The study suggested that antibiotic prophylaxis was not associated with a reduced risk of UTIs (adjusted odds ratio 0.93, 95% CI 0.45-1.94) [24]. In another study by Rianthavorn and Phithaklimnuwong, 80 neonates aged 7-30 days with AHN and mild to moderate isolated hydronephrosis (IH) on neonatal renal ultrasound were recruited from 2015 to 2016 [25]. Neonates were assigned randomly to CAP until hydronephrosis resolution (CAP group, n=40) or to watchful observation (control group, n=40) [25]. Only 34 patients received CAP due to nonadherence [25]. UTI occurred in 5 out of 34 (14.7%) patients in the CAP group versus 4 out of 40 (10.0%) patients in the control group [25]. The risk of UTI was increased in the CAP group, and UTI caused by cotrimoxazole resistant bacteria was found to be four times higher in the CAP vs. control group [25]. The trial was terminated prematurely due to the negative impact of CAP on bacterial sensitivity [25]. The authors suggested that the benefits of CAP in infants with mild to moderate IH were inconclusive, and CAP conferred a high risk of resistant bacterial organisms when UTI occurs [25].

We reviewed five articles in which the role of ABP was evaluated in the setting of HN. Most of our studies showed a minimal benefit of ABP; however, the studies had limitations in terms of disease severity, premature end of the trial, and sample size. Research conducted by Braga et al. showed the benefit of CAP in high-grade HN, and research conducted by Herz et al. showed the benefit of CAP in the setting of ANH with independent risk factors like high-grade VUR, UVJ obstruction and ureteral dilation [21,22]. Currently, the majority of pediatric urologists prefer to give ABP, if HN is severe and associated with high-grade VUR, to prevent UTIs and related complications in children.

VUR and Antibiotic Prophylaxis

The pharmacological treatment of VUR is aimed at the prevention of recurrent UTI. Current evidence suggests that CAP reduces the rate of recurrence of UTI until the reflux resolves. The majority of lower-grade VURs spontaneously resolve as the child grows [26]. Roussey-Kessler et al. performed a randomized controlled trial (RCT) of 225 children in which children from ages one month to three years with grade I-III VUR were assigned randomly, to receive daily cotrimoxazole or no treatment and followed up for 18 months [27]. The distribution of gender, VUR grade, and age of participants was similar between the two groups [27]. A significant difference was not observed in the incidence of UTIs between the two groups (17% vs. 26%, p = 0.2); however, a significant association was found between the treatment and patient gender (p = 0.017) [27]. Prophylaxis significantly reduced UTI in boys (p = 0.013), most notably in boys with grade III VUR (p = 0.042) [27]. The Swedish reflux trial randomized 203 children (128 females and 75 males aged 1-2 years) with VUR grade III-IV and assigned them to three management options: CAP, endoscopic treatment, or surveillance [28]. It was concluded that CAP and endoscopic treatment were associated with a decreased rate of UTIs in girls: 8/43 (19%) on CAP, 10/43 (23%) on endoscopic treatment, and 24/42 (57%) on surveillance (p = 0.0002) [28]. In contrast, boys did not seem to benefit from active treatment [28]. Craig et al. performed an RCT of 576 children (64% girls, 36% males with a mean age of 14 months) [29]. During the study, UTI developed in 36/288 (13%) in the group receiving CAP and in 55/288 (19%) in the placebo group [29]. The study concluded that long-term, low-dose trimethoprim/sulfamethoxazole (TMP/SMX) was associated with a decreased number of UTI in predisposed children [29]. The Randomized Intervention for Children With Vesicoureteral Reflux (RIVUR) study was a large multicenter, randomized placebo-controlled trial that included 607 children (558 females and 49 males) aged 2-71 months with grade I-IV reflux [30]. It evaluated the efficacy of ABP in preventing UTI recurrences (primary outcome) [30]. Secondary outcomes were renal scarring, treatment failure, and antimicrobial resistance [30]. Recurrent UTI developed in 39/302 children who received ABP, compared to 72/305 children who received placebo (relative risk 0.55; 95% CI 0.38-0.78) [30]. The results of the RIVUR trial showed that ABP reduced the risk of recurrent UTI by 50% (95% CI 0.34-0.74, hazard ratio 0.50) [30]. However, the occurrence of renal scarring did not differ significantly between the prophylaxis and placebo groups (11.9% and 10.2%, respectively) [30].

In a study by Pennesi et al., 100 patients with VUR (grade II, III, or IV) diagnosed with cystourethrography after the first episode of acute pyelonephritis were assigned randomly to receive ABP with TMP-SMX, or no ABP for two years [31]. The baseline characteristics of the two study groups were similar [31]. At the end of follow-up, the occurrence of renal scars was the same in children who received ABP versus those who did not receive ABP [31]. Montini et al. randomized 338 children (234 girls and 104 boys, from ages two months to less than seven years) after the first episode of febrile UTI to either CAP group (co-trimoxazole or co-amoxiclav) or no CAP group for 12 months [32]. The intention-to-treat analysis showed no significant difference in the recurrence of febrile UTIs between CAP (15/211, 7%) and no CAP (12/127, 9%) groups [32]. In the subgroup of children with reflux, the recurrence of febrile UTIs was 19.6% (9/46) on no prophylaxis and 12.1% (10/82) on prophylaxis [32]. The study concluded that CAP did not decrease the risk of recurrent febrile UTIs in children with or without low-grade VUR [32]. Hari et al. randomized 93 children (31 girls and 62 boys, median age 4.6 years) with VUR grade I-IV (73% with grade III-IV VUR) to either receive co-trimoxazole or placebo for 12 months [33]. One symptomatic UTI occurred in 10 patients receiving ABP, and in three patients receiving placebo [33]. Compared to the placebo group, the antibiotic group had a 14.8% increased risk for developing UTI [33]. In the renal scan done at 12 months, it was found that 16.2% (6/37) patients in the antibiotic group and 16.3% (7/43) patients in the placebo group had new or worsening of pre-existing scars [33]. The study concluded that long-term ABP with TMP-SMX is associated with an increased risk of symptomatic UTI compared to placebo in children with grade I-IV VUR [33]. Garin et al. selected patients between 3 months and 18 years of age with acute pyelonephritis, with or without VUR (mild/moderate only), and assigned them randomly to receive urinary antibiotic prophylaxis or not [34]. The patients were followed up every three months for one year [34]. Among the 236 patients enrolled in the study, 218 completed the one-year follow-up [34]. No statistically significant difference was noted among groups, in terms of rate of recurrent UTI, subsequent pyelonephritis, and development of renal scars [34]. This study does not support the role of ABP in preventing the recurrence of infection and the development of renal scars [34].

We reviewed eight RCTs published between 2006 and 2014, comparing CAP to no CAP in children with VUR. Four RCTs demonstrated that the use of CAP in the setting of VUR leads to an overall reduction in UTI. In contrast, four RCTs examined the use of CAP in children with VUR and did not show any statistically significant benefit of CAP in terms of reduction of UTIs, acute pyelonephritis, and renal parenchymal scarring.

Ureterocele and Antibiotic Prophylaxis

Since ureterocele is frequently associated with HN and VUR, the aim of ABP is to prevent the occurrence of UTI before surgery. Few trials have been done to determine the efficacy of CAP in the management of ureterocele. Besson et al. evaluated the incidence of neonatal UTI in the presence of a ureterocele [35]. Fifteen patients with antenatally detected ureteroceles were reviewed [35]. Among the 15 patients, eight already had a UTI on admission, and three of them had received prophylactic antibiotics [35]. The study concluded that, if diagnosed antenatally, obstruction due to ureterocele should be relieved as early as possible during the first week of life, as antibiotic prophylaxis alone was not sufficient to avoid UTIs [35]. In another study by Husmann et al., infants younger than two weeks with an extravesical ureterocele associated with a duplex upper pole moiety were assigned to either immediate endoscopic puncture of the ureterocele followed by ABP, or ABP with plans for delayed surgical intervention [36]. No statistical difference was noted between the treatment groups [36].

The studies we reviewed showed a limited benefit of ABP in the setting of ureterocele; however, the trends followed by pediatric urologists still favor the use of ABP until the resolution of disease. Table 1 presents the summary of studies from the review.

**Table 1:** Selected studies included in the review CAKUT, congenital anomalies of the kidney and urinary tract; ABP, antibiotic prophylaxis; HN, hydronephrosis; UTI, urinary tract infection; CAP, continuous antibiotic prophylaxis; ANH, antenatal hydronephrosis; VUR, vesicoureteral reflux; UVJ, ureterovesical junction; TMP-SMX, trimethoprim/sulfamethoxazole

Clinical studies	CAKUT	Sample size (N)	Benefit of ABP	Notes
Braga et al. [21]	HN	3876 infants	Yes	ABP reduces the rate of UTI with high-grade HN.
Herz et al. [22]	HN	507 children (405 after exclusion; 236 girls and 169 boys)	Yes	CAP reduces the risk of febrile UTI in children having independent risk factors (i.e., high-grade VUR, UVJ obstruction and ureteral dilation) with ANH.
Varda et al. [23]	HN	594 infants (365 males, 135 females)	No	Use of CAP of limited benefit.
Zareba et al. [24]	HN	376 (277 males, 99 females)	No	ABP not associated with a decreased risk of UTI.
Rianthavorn and Phithaklimnuwong [25]	HN	80 neonates	No	CAP benefits in mild to moderate isolated hydronephrosis were inconclusive.
Roussey-Kessler et al. 27]	VUR	225 (156 females, 69 males)	Yes	Prophylaxis significantly reduced UTI in boys (p = 0.013), most notably in boys with grade III VUR.
Brandström et al. [28]	VUR	203 children (128 females, 75 males)	Yes	CAP and endoscopic treatment reduced the rates of UTIs and renal scarring in girls; boys did not benefit from active treatment.
Craig et al. [29]	VUR	576 children (64% females, 36% males)	Yes	Long-term, low-dose TMP-SMX was associated with a decreased number of UTI in predisposed children.
RIVUR Trial Investigators [30]	VUR	607 children (558 females, 49 males)	Yes	ABP was associated with a substantially reduced risk of UTI recurrence, but not of renal scarring.
Penessi et al. [31]	VUR	100 patients (52 females, 48 males)	No	The presence of renal parenchymal scars was the same in children with and without ABP.
Montini et al. [32]	VUR	338 children (234 females, 104 males)	No	ABP did not reduce the risk of recurrent febrile UTIs in children with or without low-grade VUR.
Hari et al. [33]	VUR	93 children (31 females, 62 males)	No	Long-term ABP with TMP-SMX is associated with an increased risk of symptomatic UTI compared to placebo.
Garin et al. [34]	VUR	236 (218 completed the one-year follow-up)	No	The study does not support the role of ABP in preventing the recurrence of infection and the development of renal scars.
Besson et al. [35]	Ureterocele	15 infants	No	ABP alone not sufficient to prevent UTI.
Hussman et al. [36]	Ureterocele	72 infants	No	No statistical difference noted between treatment groups (endoscopic puncture vs. delayed open surgery and ABP).

Risks of Prophylactic Antibiotics

Providers should also be aware of the suggested but uncertain effects of long-term antibiotic exposure to children [10]. The benefits of the CAP must be weighed against the proven risk of increased bacterial resistance and drug-related side effects. The use of antibiotics in infancy has been associated with drawbacks and complications, some of which are associated with chronic use and some with short-term exposure during prophylaxis. CAP consists of prescribing daily antibiotics at one-quarter to one-half of the usual therapeutic dose [20]. Nitrofurantoin, TMP/SMX, amoxicillin, and cephalosporins are the antibiotics most commonly used in the prevention of UTI, but these medications have adverse reactions in children. Common adverse reactions associated with nitrofurantoin are gastrointestinal disturbance, and cutaneous reactions such as urticaria and maculopapular rash [20]. Adverse reactions related to TMP/SMX are almost exclusively due to the sulfamethoxazole component, most commonly cutaneous reactions [20]. However, serious side effects are rare and mostly reversible with the discontinuation of therapy [20]. Antibiotic exposure during the first year of life is associated with an increased risk of developing atopic diseases, including eczema, wheeze, asthma, and allergy later in life [6]. The underlying mechanism has been summarized in the so-called hygiene hypothesis, which suggests that growing up in an increasingly hygienic environment with reduced microbial exposure might increase atopic immune responses [6]. Another significant drawback to CAP is antibiotic resistance. The RIVUR trial demonstrated a substantial amount of resistance to TMP/SMX in patients on prophylaxis with breakthrough UTI compared to the patients on placebo [26].

Limitations

The clinical studies were not stratified based on gender, circumcision status, choice of antibiotic, VUR and HN grading, as well as additional independent risk factors. Some studies had limited statistical power, and some studies are more than 10 years old.

## Conclusions

The role of ABP in the management of HN, VUR, and ureterocele is still controversial. The main aim of ABP is to prevent UTIs in the pediatric population at risk, and in infants diagnosed with HN, VUR, ureterocele, and other CAKUT. Some benefit of ABP was shown in patients affected by high-grade HN and VUR. In contrast, studies that included mild HN and low-grade VUR did not show benefit with the long-term use of CAP. Limited data is available on the use of CAP, in the setting of ureterocele. The number of possible cofounders limits the generalizability of the findings. No definitive guidelines currently exist. Conducting randomized, placebo-controlled trials with proper standardization and stratification is likely to be essential for developing clear guidelines.

## References

[1] Abdou MSM, Sherif AAR, Wahdan IMH, Ashour KSED (2019). Pattern and risk factors of congenital anomalies in a pediatric university hospital, Alexandria, Egypt. J Egypt Public Health Assoc.

[2] Vivante A, Kohl S, Hwang DY, Dworschak GC, Hildebrandt F (2014). Single-gene causes of congenital anomalies of the kidney and urinary tract (CAKUT) in humans. Pediatr Nephrol.

[3] Yosypiv IV (2012). Congenital anomalies of the kidney and urinary tract: a genetic disorder?. Int J Nephrol.

[4] Schultza K, Todab LY (2016). Genetic basis of ureterocele. Curr Genomics.

[5] Liu DB, Armstrong WR III, Maizels M (2014). Hydronephrosis: prenatal and postnatal evaluation and management. Clin Perinatol.

[6] Castagnetti M, Cimador M, Esposito C, Rigamonti W (2012). Antibiotic prophylaxis in antenatal nonrefluxing hydronephrosis, megaureter and ureterocele. Nat Rev Urol.

[7] Iqbal S, Raiz I, Faiz I (2017). Bilateral hydroureteronephrosis with a hypertrophied,trabeculated urinary bladder. Malays J Med Sci.

[8] Wolnicki M, Aleksandrovych V, Gil A, Pasternak A, Gil K (2019). Relation between ureteral telocytes and the hydronephrosis development in children. Folia Med Cracov.

[9] Easterbrook B, Capolicchio JP, Braga LH (2017). Antibiotic prophylaxis for prevention of urinary tract infections in prenatal hydronephrosis: an updated systematic review. Can Urol Assoc J.

[10] Lee T, Park JM (2017). Vesicoureteral reflux and continuous prophylactic antibiotics. Investig Clin Urol.

[11] Wu CQ, Franco I (2017). Management of vesicoureteral reflux in neurogenic bladder. Investig Clin Urol.

[12] Choi YH, Cheon JE, Kim WS, Kim IO (2016). Ultrasonography of hydronephrosis in the newborn: a practical review. Ultrasonography.

[13] Shah H, Tiwari C, Shenoy NS, Dwivedi P, Gandhi S (2017). Transurethral incision of ureteroceles in paediatric age group. Turk J Urol.

[14] Turkyilmaz G, Cetin B, Sivrikoz T (2019). Antenatally detected ureterocele: associated anomalies and postnatal prognosis. Taiwan J Obstet Gynecol.

[15] Shokeir AA, Nijman RJ (2002). Ureterocele: an ongoing challenge in infancy and childhood. BJU Int.

[16] Leung AKC, Wong AHC, Leung AAM, Hon KL (2019). Urinary tract infection in children. Recent Pat Inflamm Allergy Drug Discov.

[17] Wong NC, Koyle MA, Braga LH (2017). Continuous antibiotic prophylaxis in the setting of prenatal hydronephrosis and vesicoureteral reflux. Can Urol Assoc J.

[18] Visuri S, Jahnukainen T, Taskinen S (2017). Incidence of urinary tract infections in infants with antenatally diagnosed hydronephrosis - a retrospective single center study. J Pediatr Surg.

[19] Zee RS, Herbst KW, Kim C (2016). Urinary tract infections in children with prenatal hydronephrosis: a risk assessment from the Society for Fetal Urology Hydronephrosis Registry. J Pediatr Urol.

[20] Song SH, Kim KS (2008). Antibiotic prophylaxis in pediatric urology. Indian J Urol.

[21] Braga LH, Mijovic H, Farrokhyar F, Pemberton J, DeMaria J, Lorenzo AJ (2013). Antibiotic prophylaxis for urinary tract infections in antenatal hydronephrosis. Pediatrics.

[22] Herz D, Merguerian P, McQuiston L (2014). Continuous antibiotic prophylaxis reduces the risk of febrile UTI in children with asymptomatic antenatal hydronephrosis with either ureteral dilation, high-grade vesicoureteral reflux, or ureterovesical junction obstruction. J Pediatr Urol.

[23] Varda BK, Finkelstein JB, Wang HH, Logvinenko T, Nelson CP (2018). The association between continuous antibiotic prophylaxis and UTI from birth until initial postnatal imaging evaluation among newborns with antenatal hydronephrosis. J Pediatr Urol.

[24] Zareba P, Lorenzo AJ, Braga LH (2014). Risk factors for febrile urinary tract infection in infants with prenatal hydronephrosis: comprehensive single center analysis. J Urol.

[25] Rianthavorn P, Phithaklimnuwong S (2020). The role of antibiotic prophylaxis in mild to moderate isolated hydronephrosis detected in antenatal screening. Investig Clin Urol.

[26] Johnston DL, Qureshi AH, Irvine RW, Giel DW, Hains DS (2016). Contemporary management of vesicoureteral reflux. Curr Treat Options Pediatr.

[27] Roussey-Kesler G, Gadjos V, Idres N (2008). Antibiotic prophylaxis for the prevention of recurrent urinary tract infection in children with low grade vesicoureteral reflux: results from a prospective randomized study. J Urol.

[28] Brandström P, Jodal U, Sillén U, Hansson S (2011). The Swedish reflux trial: review of a randomized, controlled trial in children with dilating vesicoureteral reflux. J Pediatr Urol.

[29] Craig JC, Simpson JM, Williams GJ (2010). Antibiotic prophylaxis and recurrent urinary tract infection in children [published correction appears in N Engl J Med. 2010, 362:1250]. N Engl J Med.

[30] RIVUR Trial Investigators, Hoberman A, Greenfield SP (2014). Antimicrobial prophylaxis for children with vesicoureteral reflux. N Engl J Med.

[31] Pennesi M, Travan L, Peratoner L (2008). Is antibiotic prophylaxis in children with vesicoureteral reflux effective in preventing pyelonephritis and renal scars? A randomized, controlled trial. Pediatrics.

[32] Montini G, Rigon L, Zucchetta P (2008). Prophylaxis after first febrile urinary tract infection in children? A multicenter, randomized, controlled, noninferiority trial. Pediatrics.

[33] Hari P, Hari S, Sinha A, Kumar R, Kapil A, Pandey RM, Bagga A (2015). Antibiotic prophylaxis in the management of vesicoureteric reflux: a randomized double-blind placebo-controlled trial. Pediatr Nephrol.

[34] Garin EH, Olavarria F, Garcia Nieto V, Valenciano B, Campos A, Young L (2006). Clinical significance of primary vesicoureteral reflux and urinary antibiotic prophylaxis after acute pyelonephritis: a multicenter, randomized, controlled study. Pediatrics.

[35] Besson R, Ngoc BT, Laboure S, Debeugny P (2000). Incidence of urinary tract infection in neonates with antenatally diagnosed ureteroceles. Eur J Pediatr Surg.

[36] Husmann DA, Strand WR, Ewalt DH, Kramer SA (2002). Is endoscopic decompression of the neonatal extravesical upper pole ureterocele necessary for prevention of urinary tract infections or bladder neck obstruction?. J Urol.

